# Solitary Bone Plasmacytoma of the Scapula: A Rare Localization With Remarkable Radiotherapy Outcome

**DOI:** 10.7759/cureus.98499

**Published:** 2025-12-05

**Authors:** Reyzane El Mjabber, Rim Alami, Ech-Cherki El Mjabber, Zineb Dahbi, Fadila Kouhen, Nabil Ismaili, Sanaa El Majjaoui, Asmaa Naim

**Affiliations:** 1 Radiation Oncology, Mohammed VI University of Sciences and Health (UM6SS), Casablanca, MAR; 2 Radiation Oncology, International University Cheikh Khalifa Hospital, Casablanca, MAR; 3 Radiation Oncology, Mohammed VI University of Sciences and Health (UM6SS), Casablanca, MAR; 4 Orthopaedics and Trauma, Clinique AL Marjane, Khouribga, MAR; 5 Medical Oncology, Mohammed VI University of Sciences and Health (UM6SS), Casablanca, MAR; 6 Medical Oncology, International University Cheikh Khalifa Hospital, Casablanca, MAR

**Keywords:** local control, monoclonal plasma cells, radiotherapy, rare localization, solitary bone plasmacytoma

## Abstract

Solitary bone plasmacytoma (SBP) is a rare plasma cell neoplasm characterized by localized proliferation of monoclonal plasma cells in bone without systemic involvement, which distinguishes it from multiple myeloma. Although the axial skeleton is most frequently affected, involvement of the shoulder girdle is exceptionally uncommon.

We present the case of a 48-year-old woman who experienced progressive left shoulder pain and noted an enlarging mass over six months. Imaging revealed a large osteolytic lesion in the scapula with extension into surrounding soft tissue. Histopathology and immunohistochemistry confirmed the diagnosis of solitary plasmacytoma. Comprehensive staging, including bone marrow biopsy and 18F-FDG PET/CT (Fluorine-18 fluorodeoxyglucose positron emission tomography/computed tomography), excluded systemic disease. The patient underwent definitive radiotherapy, receiving 50 Gy (Grays) in 25 fractions via a 3D (three-dimensional) conformal technique with careful target delineation. Treatment was well tolerated, with only grade I radiodermatitis reported. Follow-up imaging two months after therapy demonstrated significant lesion regression, partial re-ossification, and reduction of soft-tissue involvement.

SBP of the scapula represents a rare clinical entity, and radiotherapy continues to be the treatment of choice, offering excellent local control. Despite favorable local outcomes, long-term monitoring is crucial due to the potential risk of progression to multiple myeloma. Documenting rare localizations contributes to a better understanding of disease behavior and supports tailored management approaches.

## Introduction

The solitary plasmacytoma (SP) is a rare malignant tumor characterized by a clonal proliferation of monoclonal plasma cells, without associated systemic involvement, unlike multiple myeloma. It accounts for less than 5% of plasma cell neoplasms and is distinguished by its unique localization, either in bone (solitary bone plasmacytoma [SBP]) or in soft tissues (extramedullary plasmacytoma [EMP]).

Clinically, SBP presents symptoms related to the local tumor mass: pain, swelling, and pathological fractures. The absence of systemic signs such as anemia, hypercalcemia, or renal failure distinguishes SP from multiple myeloma and can sometimes make the diagnosis challenging.

The diagnosis is based on histology, confirming the monoclonal plasma cell proliferation, and on a comprehensive work-up aimed at excluding systemic involvement. Modern imaging, particularly PET-CT (positron emission tomography-computed tomography), allows for the assessment of tumor extent and the detection of possible multiple lesions, improving diagnostic accuracy and follow-up. Solitary plasmacytoma carries a variable risk of progression to multiple myeloma. Radiotherapy is the treatment of choice, with curative doses of 40-50 Gy, while surgery is reserved for cases requiring decompression or bone stabilization.

We present a case of a solitary plasmacytoma of the scapula successfully managed with radiotherapy, along with a comprehensive review of the literature.

## Case presentation

A 48-year-old female patient with an unremarkable medical history presented with a six-month history of persistent, progressively worsening pain in the left shoulder, initially without limitation of movement, which later became associated with restricted arm elevation and the gradual development of an enlarging mass in the posteroinferior region of the left shoulder.

The patient denied any history of direct or indirect trauma to the shoulder or any prior radiation exposure. Further history revealed no recent illnesses and no history of substance abuse, including alcohol and tobacco. Her family history was negative for malignancies.

Her overall condition remained stable, with no fever, unintentional weight loss, or loss of appetite.

Despite the use of over-the-counter analgesics, the shoulder pain persisted and progressively intensified, ultimately disrupting her sleep, prompting her to seek medical attention.

The patient initially consulted a traumatologist, who found a 9-cm firm, irregular, and fixed mass on the left shoulder, exquisitely painful on palpation. The overlying skin appeared normal, without erythema or ulceration. Musculoskeletal examination revealed significant limitations of shoulder movements: flexion 80°, extension 20°, abduction 50°, adduction 20°, external rotation 10°, and internal rotation 30°.

Deep tendon reflexes remained intact, and there was no evidence of peripheral nerve involvement in the left upper limb.

**Figure 1:**
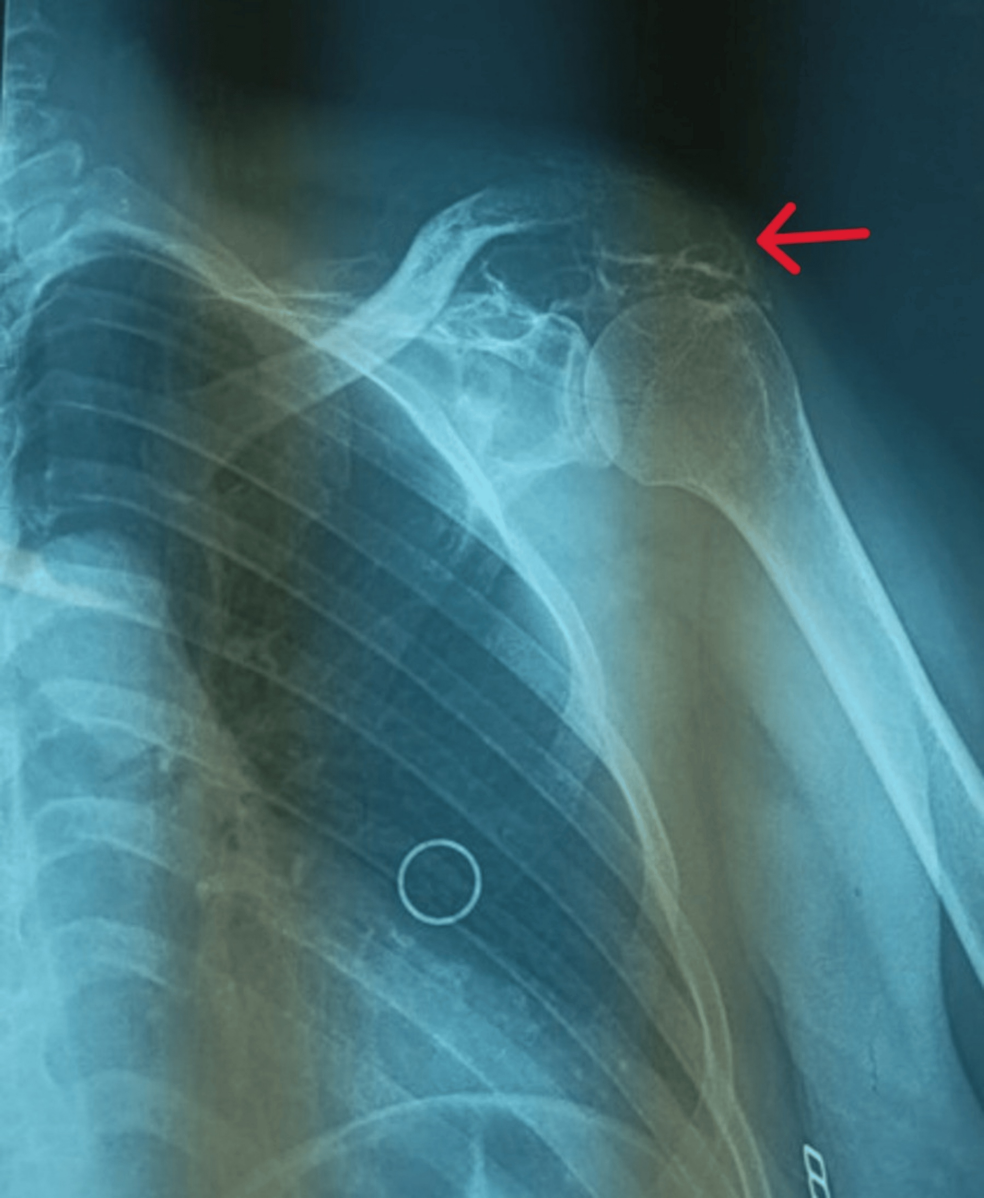
Frontal radiograph showing a solitary osteolytic lesion in the posteroinferior scapula with irregular, slightly scalloped margins and mild cortical thinning (arrow). The glenohumeral joint is preserved.

A frontal (anteroposterior) radiography of the left shoulder was performed, finding a solitary, localized osteolytic lesion in the posteroinferior aspect of the scapula, corresponding to the clinically palpable mass. The lesion demonstrates irregular, slightly scalloped margins with a lytic appearance, and mild cortical thinning is noted in some areas. No overt pathological fracture is identified. The glenohumeral joint is maintained, with no evidence of dislocation or significant articular surface involvement. The proximal humerus appears unremarkable. Evaluation of adjacent soft tissues is limited on this radiograph, though there may be a subtle prominence corresponding to the lesion (Figure 1).

**Figure 2:**
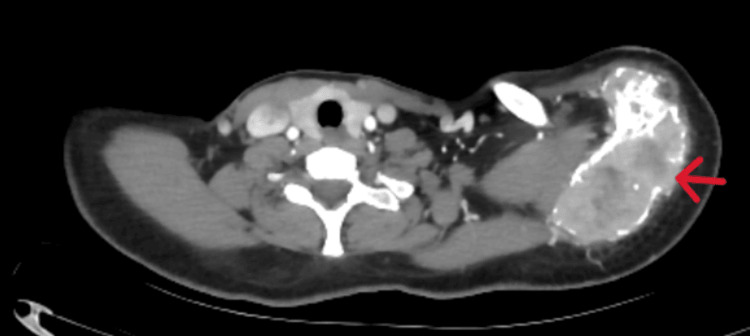
Axial CT scan of the shoulder showing heterogeneous enhancement after contrast injection, with preservation of the vascular bundle.

**Figure 3:**
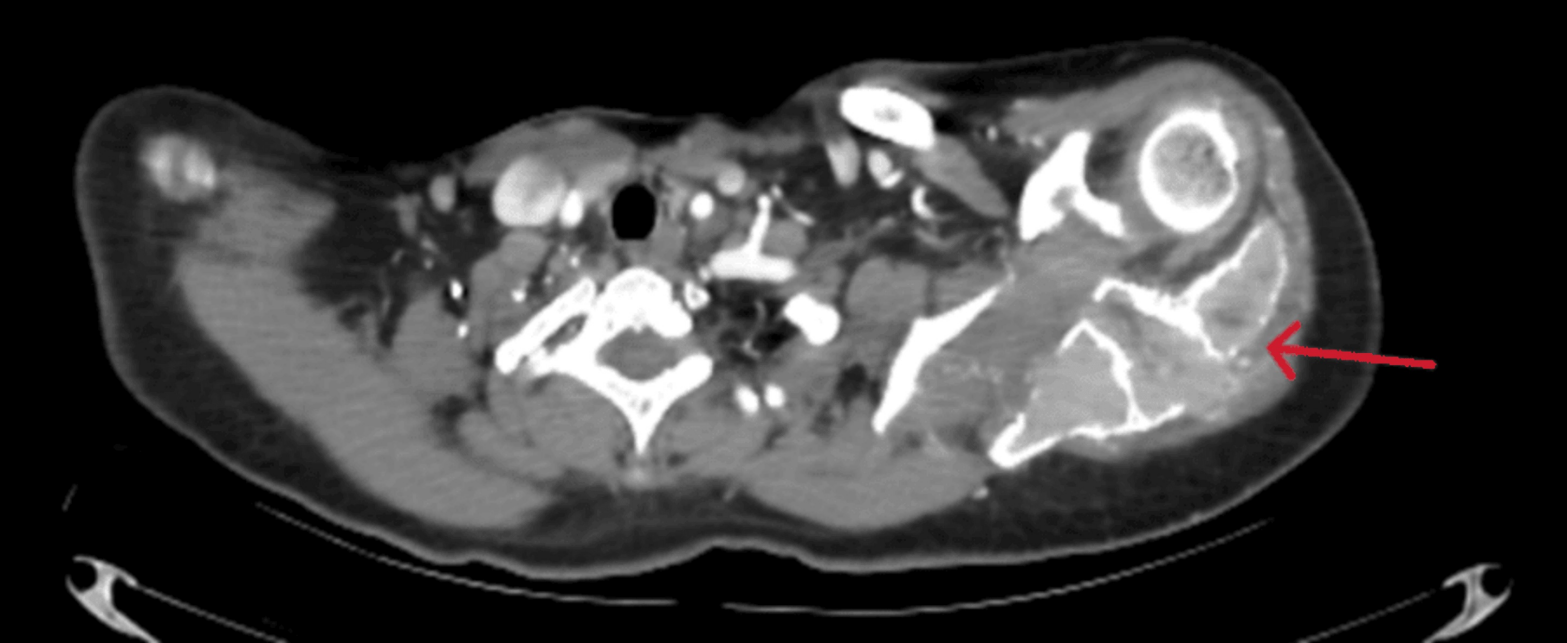
Axial CT scan of the shoulder showing a lesion with irregular contours and cortical bone disruption, extending into the adjacent soft tissues near the deltoid muscle (arrow).

**Figure 4:**
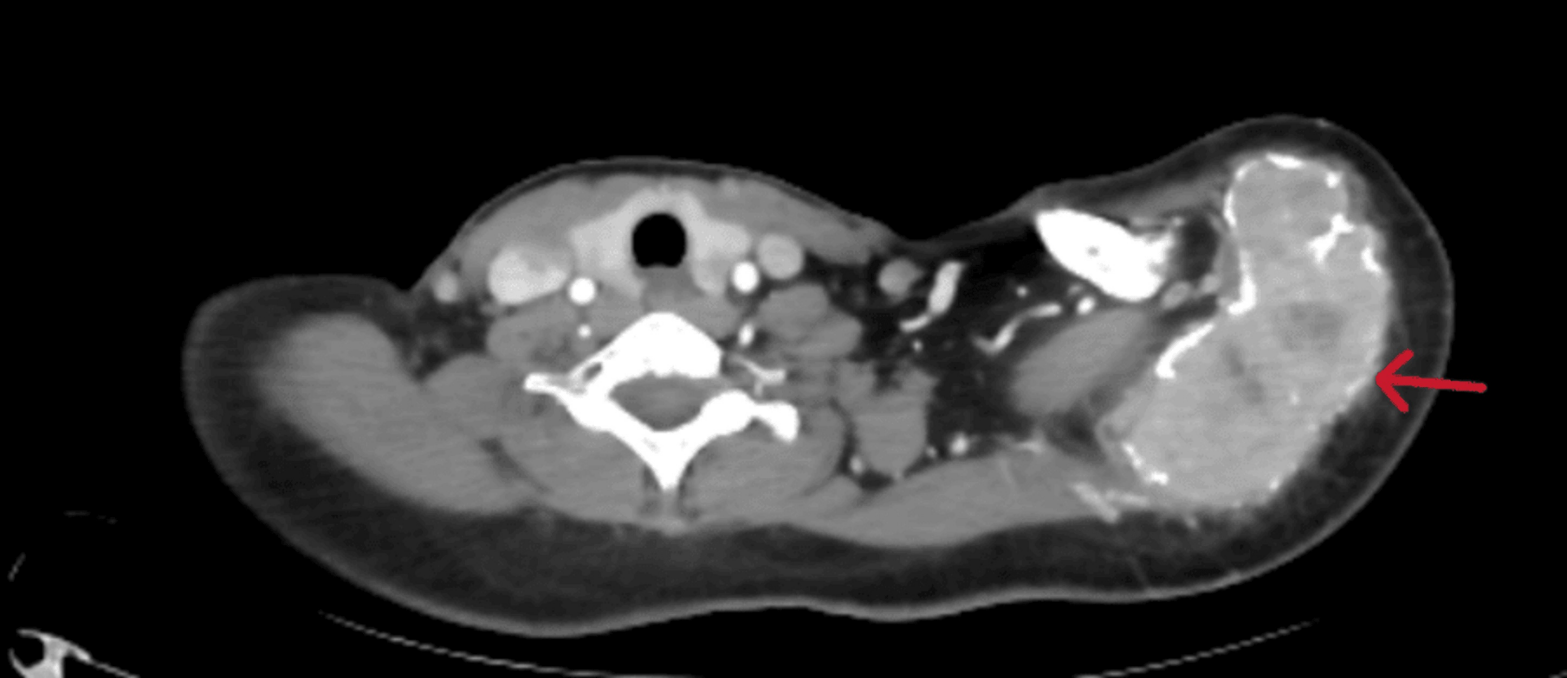
Axial CT scan of the shoulder showing a mass with irregular borders and cortical breakthrough (arrow).

A CT scan of the left shoulder was subsequently requested to more accurately assess the extent of the lesion and the involvement of adjacent soft tissues, finding a large mass centered on the acromion, extending to the spine of the left scapula and developing in the supraspinous fossa. The lesion has irregular contours, tissue density, and an osteolytic nature with a layered appearance and cortical bone rupture, extending into the adjacent soft tissues near the deltoid muscle. The lesion shows intense and heterogeneous enhancement after intravenous injection of iodinated contrast. It measures approximately 90 x 57 mm with a craniocaudal extension of 71 mm. The humeral vascular bundle remains at a distance and is patent. The skin covering is thin and regular (Figures 2-4).

**Figure 5:**
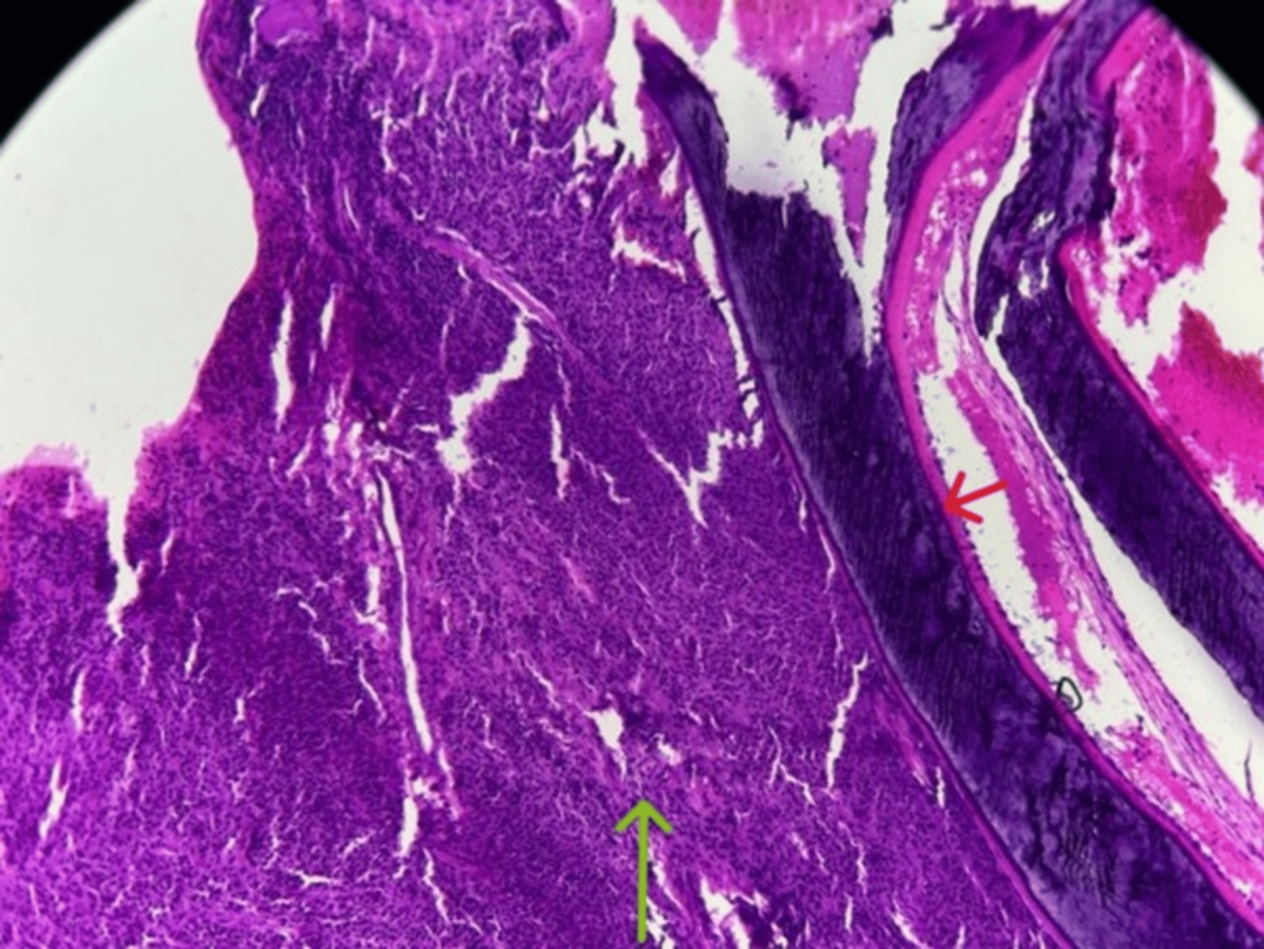
Hematoxylin and eosin stain, ×10 magnification. The tumor consists of malignant cells (green arrow) arranged in sheets and surrounded by bony trabeculae (red arrow).

**Figure 6:**
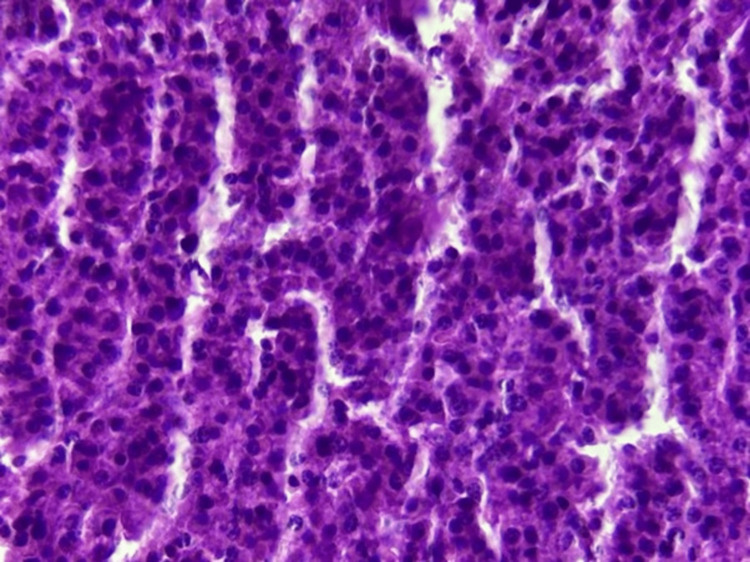
Hematoxylin and eosin stain, ×40 magnification. Malignant round-cell tumor showing small monomorphic cells and a congested capillary stroma.

The patient underwent a percutaneous biopsy from the left shoulder tumor, including both muscular and bony tissue (Figure 5), which shows a vascularized malignant proliferation composed of round cells arranged in a diffuse pattern (Figure 6). The tumor cells are small to medium-sized and monomorphic, with small nuclei displaying coarse chromatin. The stroma is scant and mainly consists of congested capillary blood vessels. These features are consistent with a malignant round-cell tumor.

**Figure 7:**
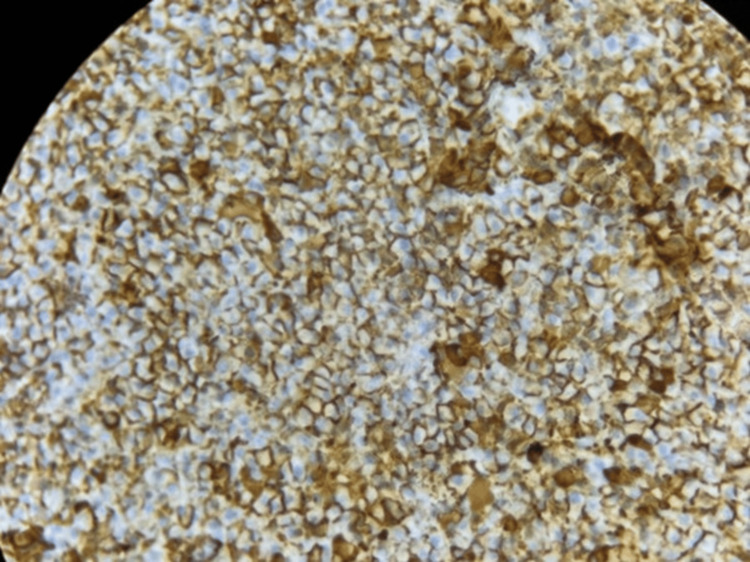
CD138 immunohistochemistry demonstrating membranous positivity in plasma cells (×40 magnification). CD: Cluster of Differentiation

**Figure 8:**
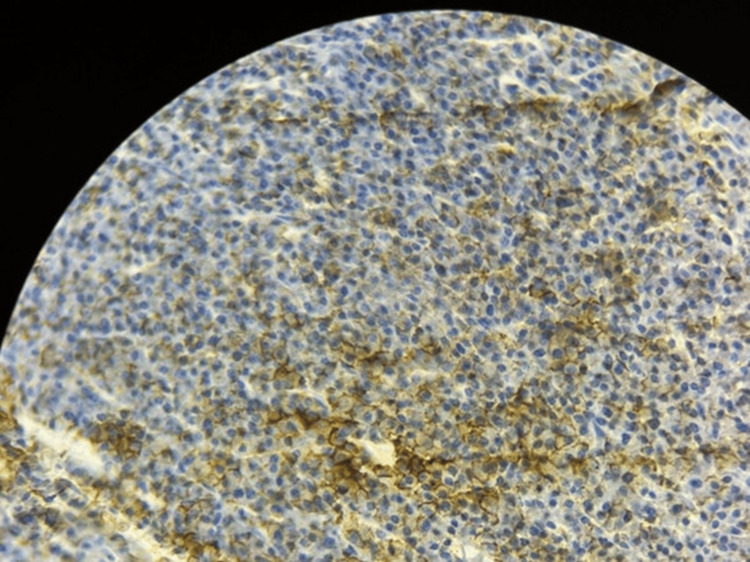
EMA immunohistochemistry demonstrating membranous positivity in tumor cells (×40 magnification). EMA: Epithelial Membrane Antigen

**Figure 9:**
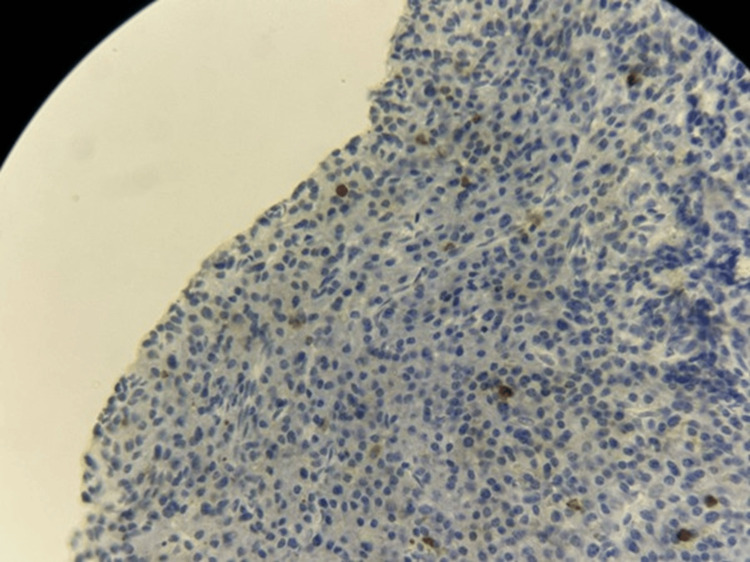
Immunohistochemistry for Ki-67 demonstrating rare positive tumor cells (×40 magnification).

**Figure 10:**
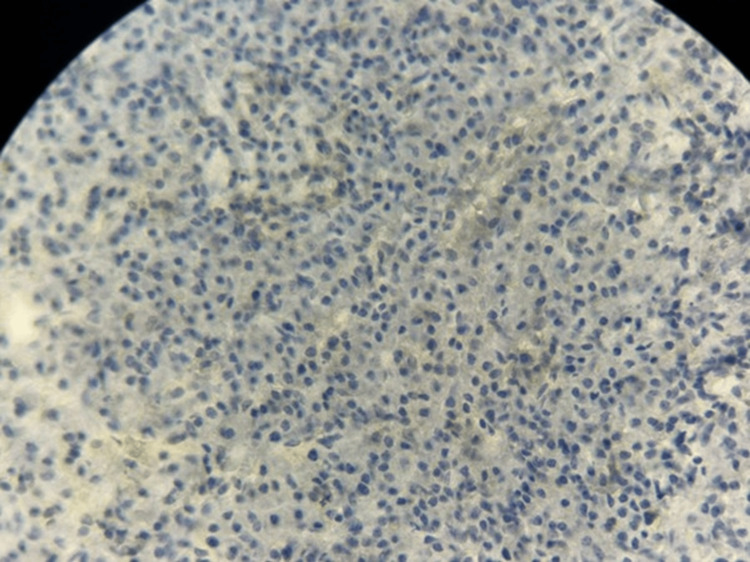
Immunohistochemistry showing absence of CD20 expression in tumor cells (×40 magnification).

**Figure 11:**
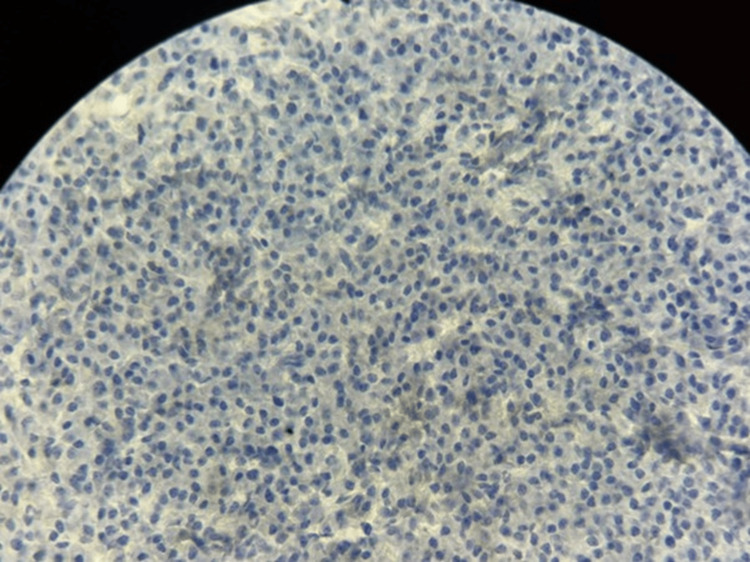
Immunohistochemistry showing absence of AE1/AE3 (pan-cytokeratin) expression in tumor cells (×40 magnification).

**Figure 12:**
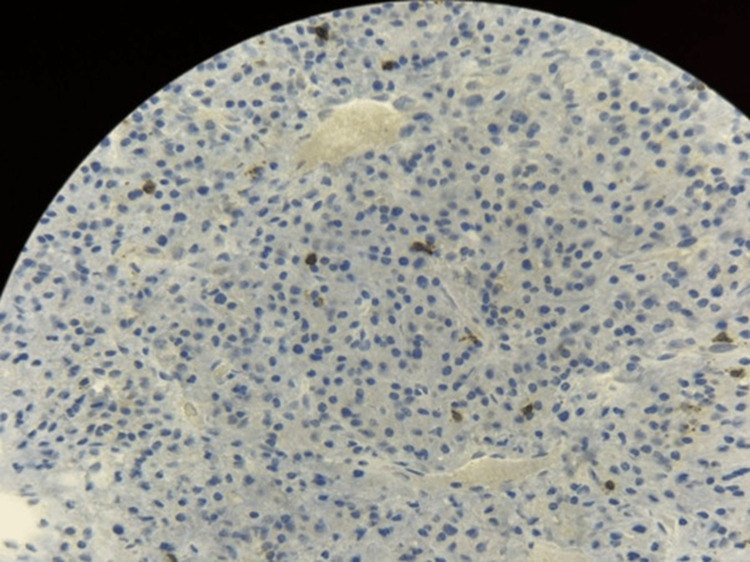
Immunohistochemistry showing rare tumor cells exhibiting CD3 (T-cell surface antigen) positivity (×40 magnification).

Immunohistochemistry (IHC) demonstrated that tumor cells were negative for anti-CD20 (antibody against CD20, a B-cell marker; Clone L26, Bio SB) and anti-pan-cytokeratin (antibody against pan-cytokeratin, an epithelial cell marker; Clone AE1/AE3, Bio SB). Anti-CD3 (antibody against CD3, a T-cell marker; Clone RBT-CD3e, Bio SB) was positive in a few scattered tumor cells. The tumor cells showed diffuse positivity for anti-CD138 (antibody against CD138, a plasma cell marker; Clone B-A38, Bio SB) and anti-EMA (antibody against epithelial membrane antigen [EMA], a membrane marker expressed in plasma and epithelial cells; Clone E29, Epredia). The proliferation index assessed by anti-Ki67 (antibody against Ki-67, a nuclear protein used to evaluate tumor proliferation; Clone SP6, Thermo Scientific) was approximately 10%. Overall, the immunohistochemical profile is consistent with a plasmacytoma (Figures 7-12).

The case was presented to a multidisciplinary team (MDT), which recommended a staging workup to search for secondary lesions in the context of multiple myeloma.

A blood workup, including a complete blood count, liver function tests, renal function tests, protein electrophoresis, and serologies, was within normal limits. Also, a urine protein electrophoresis was performed, and it did not demonstrate any detectable urinary M-protein. A bone marrow aspirate showed a hypercellular marrow with numerous megakaryocytes, a granulocyte-to-erythroid (G/E) ratio of 2, no obvious dysplasia, and no excess of blasts. Activated macrophages were noted in relatively high numbers. The marrow contained 3% plasma cells and 13% lymphocytes.

**Figure 13:**
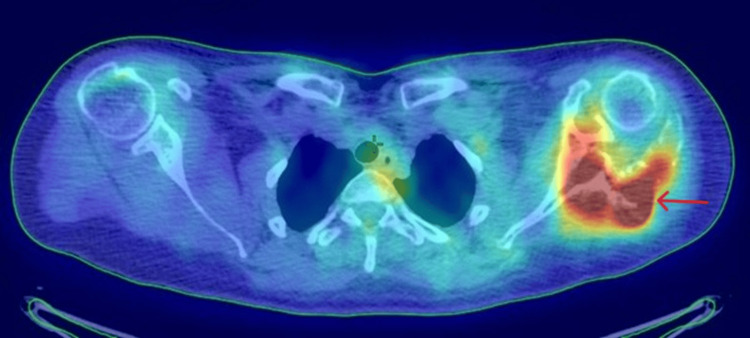
Axial view showing intense hypermetabolic activity in the left shoulder, corresponding to a lytic lesion involving the acromion and glenoid (SUVmax 19.74).

The patient underwent an 18F-FDG PET/CT (fluorine-18 fluorodeoxyglucose positron emission tomography/computed tomography) showing a homogeneous and symmetric cerebral uptake. In the cervico-thoracic region (mediastinal SUV 2.46), physiological hypermetabolism was noted in the ENT region, with no suspicious cervical or mediastinal lymph node uptake and no abnormal pleuropulmonary activity. In the abdomino-pelvic region (liver SUV 3.38), uptake in the liver, spleen, and adrenal glands was homogeneous, while gastroduodenal hypermetabolism (SUVmax 10.14) suggested inflammatory activity. No pathological uptake was observed in the abdominopelvic lymph nodes. The left shoulder demonstrated hypermetabolic activity corresponding to a lytic lesion involving the acromion and glenoid (SUVmax 19.74), with no other suspicious skeletal uptake detected elsewhere. Overall, the exam revealed hypermetabolic activity confined to the left shoulder, with no other areas of concern (Figure 13).

The case was presented at MDT for a second time with these findings, and the decision was made to proceed with radiotherapy to the shoulder.

During the simulation process, the patient was positioned supine on a Vac-Lok cushion, along with additional immobilization devices such as a headrest, to ensure consistent positioning throughout the imaging procedure and daily radiotherapy sessions. A small tattoo was placed on the patient’s shoulder to ensure accurate reproduction of the simulation position. CT scans were then acquired, ensuring comprehensive coverage of the region of interest from the calvaria to the diaphragm.

**Figure 14:**
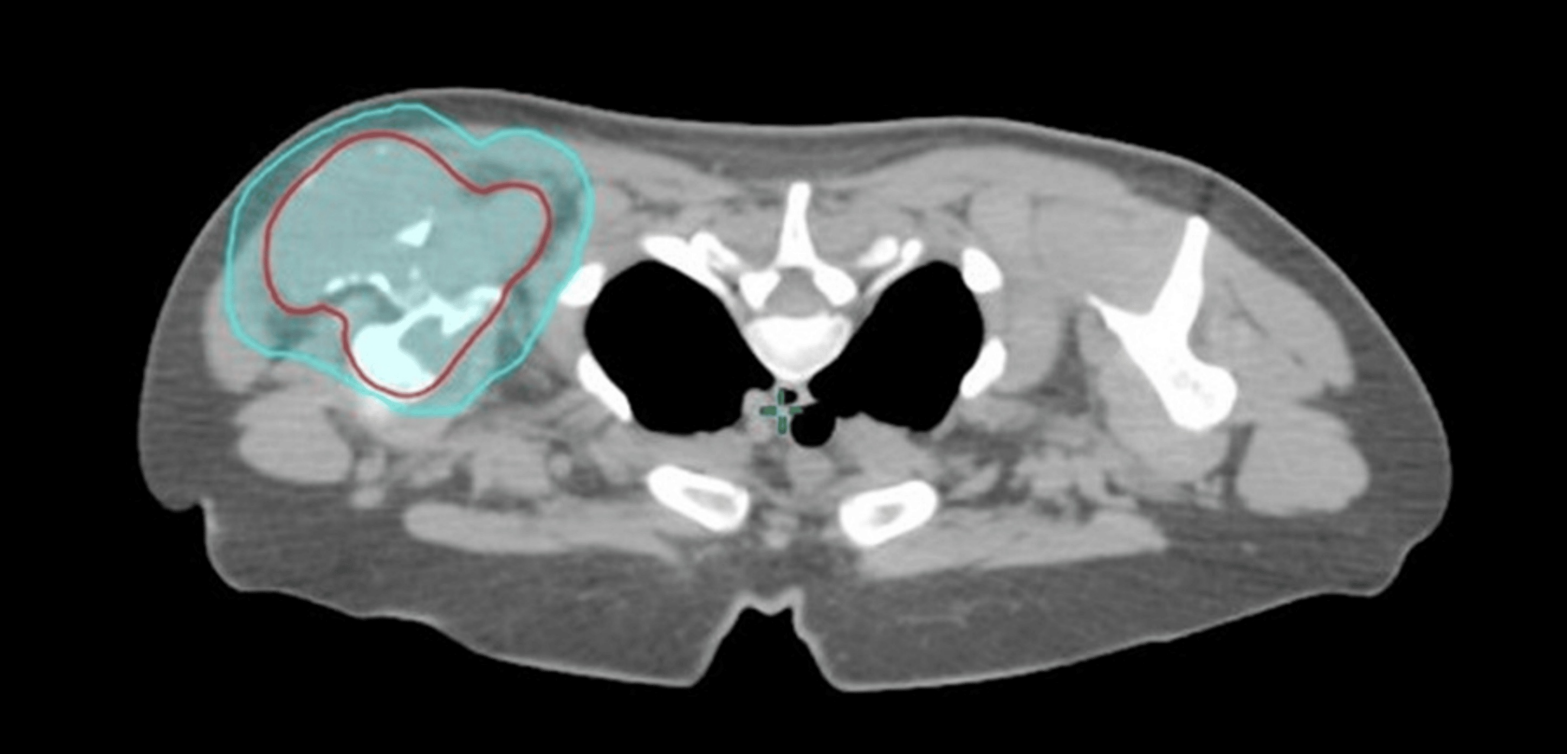
Axial CT image from the patient’s dosimetric scan showing target volume delineation with the CTV in red and the PTV in blue for the left shoulder plasmacytoma. CTV: Clinical Target Volume, GTV: Gross Tumor Volume

The planning and tumor segmentation for radiotherapy (RT) were meticulously handled by a multidisciplinary team, including a radiation oncologist, medical physicist, and dosimetrist. The radiation oncologist defined the target area and critical structures based on imaging and clinical findings to minimize healthy tissue exposure. The Gross Tumor Volume (GTV) was defined as the macroscopically visible tumor on CT, showing contrast enhancement after injection, including the lesion within the bone as well as its extension into the adjacent soft tissues. The clinical target volume (CTV), in red, encompasses the original GTV and suspected microscopic subclinical disease. In the setting of definitive radiotherapy for solitary plasmocytoma, our CTV included the GTV plus a margin of 2 cm expanded in all directions, respecting anatomic boundaries. with a 1 cm margin for the planning target volume (PTV), in blue [1] (Figure 14).

**Figure 15:**
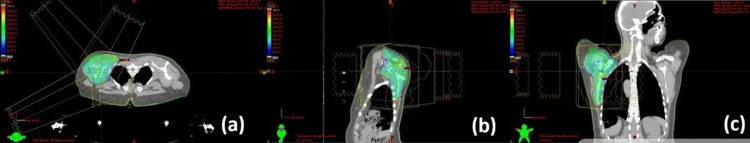
Treatment planning CT showing the 3D conformal dose distriTreatment planning CT showing the 3D conformal dose distribution for the left shoulder plasmacytoma. (a): axial view, (b): sagittal view, (c): coronal view.bution for the left shoulder plasmacytoma. (a): axial view, (b): sagital view, (c): coronal view 3D: Three-Dimensional

The medical physicist ensured accurate dose calculations and safety compliance, while the dosimetrist developed a precise treatment plan using advanced software for optimal radiation delivery. Treatment planning was conducted using the Eclipse treatment planning system (Varian Medical Systems). Radiotherapy was administered using a 6 MV photon beam delivered by the Clinac IX linear accelerator employing the 3D (three-dimensional) conformal radiotherapy. Daily imaging guided the treatment to ensure precise delivery. According to ASTRO recommendations for SBP larger than 5 cm, a prescribed dose of 50 Gy (Grays) was administered in daily 2 Gy fractions over five weeks [1] (Figures 15a-15c).

Dose-volume histograms (DVHs) were generated to evaluate the radiation dose distribution and assess the conformity of the treatment plan to the tumor while sparing surrounding healthy tissue. Specific dose constraints were set for critical structures, including the spinal cord, the lungs, the heart, and the humeral head (Table 1).

**Table 1:** Maximum (Dmax) and mean (Dmean) doses delivered to organs at risk (OARs) during radiotherapy planning

Organ at risk	Dmax (Gy)	Dmean (Gy)
Right Lung	29	2,34
Left Lung	49,55	19,16
Heart	15,73	9,34
Humoral Head	50,17	46,1
Spinal Cord	38,75	7

The maximum dose to the spinal cord was limited to 38.75 Gy, while the right and left lungs received Dmean = 2.34 Gy (mean dose) and Dmean = 19.16 Gy, respectively [2]. For the planning target volume (PTV), the treatment plan aimed to deliver 95% to 107% of the prescribed dose to ensure adequate coverage while maintaining safety for surrounding critical structures.

During radiotherapy, the patient developed grade I radiodermatitis, which was managed with healing creams and emollients, resulting in a good response.

**Figure 16:**
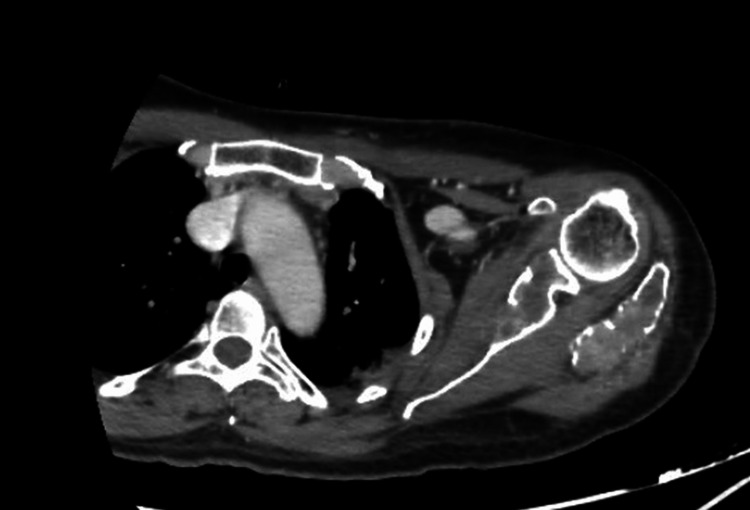
Axial follow-up CT of the shoulder showing decreased surrounding soft-tissue involvement.

**Figure 17:**
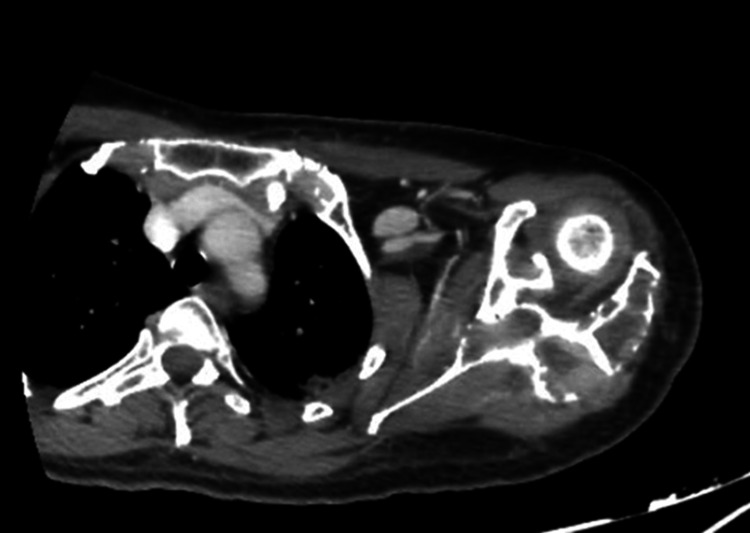
Axial follow-up CT of the shoulder showing marked reduction in the size and density of the previously observed osteolytic lesion, with partial re-ossification.

A follow-up urine protein electrophoresis was performed, and it did not demonstrate any detectable urinary M-protein. A CT scan of the shoulder was performed two months after the completion of radiotherapy, revealing a clear reduction in the extent and density of the previously observed osteolytic area, with partial re-ossification and decreased surrounding soft-tissue involvement. No new lesions or progression are identified (Figures 16, 17).

## Discussion

Solitary bone plasmacytoma (SBP) is an uncommon monoclonal plasma cell neoplasm characterized by localized proliferation of clonal plasma cells within a single bone site, without systemic features of multiple myeloma (MM) [3]. It occurs more frequently in men than in women, with a male-to-female ratio of approximately 2:1, and typically presents about a decade earlier than multiple myeloma [4]. The axial skeleton, particularly the vertebrae and skull, is most often affected, whereas involvement of long bones remains exceptional [5].

In contrast, extramedullary plasmacytoma (EMP) develops in soft tissues, most frequently in the head and neck region, especially within the nasal cavity and nasopharynx [5]. Both solitary bone plasmacytoma and extramedullary plasmacytoma differ from multiple myeloma by the absence of CRAB features, hypercalcemia, renal failure, anemia, and multiple bone lesions [4].

The diagnostic criteria for solitary bone plasmacytoma include the presence of a solitary bone lesion confirmed radiologically, histological evidence of monoclonal plasma cell infiltration, fewer than 10% plasma cells in the bone marrow, and the absence of systemic myeloma-related organ damage or serum/urine M-protein [3,4]. Conversely, extramedullary plasmacytoma is defined by tissue-based monoclonal plasma cell proliferation without osteolytic bone involvement [3]. According to Durie and Salmon, solitary bone plasmacytoma may be considered as stage I myeloma [6].

Histopathology alone is insufficient for diagnosis; a comprehensive assessment including imaging, bone marrow examination, and protein electrophoresis is mandatory to rule out multiple myeloma [3]. Fine-needle aspiration cytology (FNAC) may assist in early provisional diagnosis [7], plasma cells exhibit intracytoplasmic or extracellular crystalline inclusions composed of monoclonal immunoglobulin, which have been reported in lymphoproliferative disorders and rare cases of plasmacytoma, particularly in the parotid gland [8-10]. The prognostic value of these inclusions remains uncertain.

On radiographs, plasmacytomas appear as well-defined, “punched-out” lytic lesions with cortical destruction and marginal sclerosis [11,12]. Modern diagnostic tools such as flow cytometry and molecular analysis of heavy and light chain rearrangements allow better confirmation of clonality [3,11].

Several factors have been associated with a higher risk of progression to multiple myeloma, including lesion size greater than 5 cm, age over 40 years, vertebral localization, and persistence of M-protein after treatment [3,7,13]. Bataille and Sany reported that 15% of patients developed new solitary lesions, with 75% subsequently progressing to multiple myeloma [13].

Recent large-scale studies have refined our understanding of disease prognosis. A meta-analysis including 3,487 patients showed that minimal bone marrow infiltration (≤10% clonal plasma cells) significantly worsens disease-free survival (15.7 vs. 79 months) and that the 10-year disease-free survival rate remains around 42% [14]. Similarly, a multicenter retrospective study from the Greek Myeloma Study Group reported 5- and 10-year overall survival rates of 85% and 70%, respectively, with no clear survival benefit from adjuvant chemotherapy [15]. In Japanese cohorts, radiotherapy alone achieved high local control (≈89%), but five-year disease-free survival remained modest at 29% [16]. Furthermore, a recent SEER-based analysis highlighted socioeconomic and ethnic disparities in access to radiotherapy, which may influence outcomes in solitary bone plasmacytoma patients [17].

Radiotherapy remains the standard treatment for solitary bone plasmacytoma and provides excellent local control [3]. Given that the shoulder is a highly mobile structure affected by respiratory motion, the addition of a 1 cm margin to the PTV was considered appropriate [1]. Moreover, IMRT provides superior target coverage in cases of large tumors, where 3D conformal radiotherapy may be unable to adequately respect dose constraints for adjacent organs at risk [1].

Surgery may be indicated for stabilization of pathological fractures or decompression of neural structures. In cases with poor prognostic features, adjuvant chemotherapy can be considered [3]. Despite local control, up to two-thirds of patients eventually progress to multiple myeloma, typically within 2-3 years [3,18].

Plasmacytoma of the appendicular skeleton, such as of the shoulder, is extremely rare. Reporting such unusual presentations helps improve understanding of the biological behavior and prognostic variability of solitary bone plasmacytomas.

## Conclusions

Solitary plasmacytoma of bone represents a rare and clinically heterogeneous plasma cell disorder that requires meticulous diagnostic evaluation to distinguish it from multiple myeloma. Despite excellent local control achieved with radiotherapy, the risk of systemic progression remains significant, emphasizing the need for long-term surveillance. Advances in molecular diagnostics and imaging have improved early detection of minimal marrow involvement, which may refine prognostic stratification and guide therapeutic decisions. Given the rarity and heterogeneity of SBP, establishing multi-institutional registries would provide valuable data to better characterize clinical behavior, guide treatment selection, and strengthen evidence-based practice in this disease.
